# Inmates’ empathy: relationship with childhood victimisation

**DOI:** 10.1080/07853890.2021.1896176

**Published:** 2021-09-28

**Authors:** Renata Guarda, Telma C. Almeida

**Affiliations:** a Instituto Universitário Egas Moniz (IUEM), Egas Moniz Cooperativa de Ensino Superior, Caparica, Portugal; b Laboratório de Psicologia Egas Moniz (LabPSI), Centro de Investigação Interdisciplinar Egas Moniz (CiiEM), Egas Moniz Cooperativa de Ensino Superior, Caparica, Portugal

## Abstract

**Introduction:**

Psychological trauma can occur at any time in life [1], and when it happens during childhood or adolescence, it may have negative repercussions on mental health that prevail in adult life [2]. The main objective of this study was to evaluate the relationship between childhood victimisation trauma experiences and empathy among incarcerated males.

**Materials and methods:**

The sample was composed of 99 incarcerated males with ages between 18 and 73 years old (*M* = 37.96; *SD* = 11.6). Some of the participants were arrested for drug trafficking (*n* = 24, 24.2%), domestic violence (*n* = 10, 10.1%), homicide (*n* = 9, 9.1%), robbery (*n* = 7, 7.1%), theft (*n* = 5, 5.1%), and other types of crimes (e.g. kidnapping, attempted murder). Participants answered face-to-face to a sociodemographic questionnaire, to the Childhood Trauma Questionnaire (CTQ) [3] and the Interpersonal Reactivity Index (IRI) [4].

**Results:**

All the participants experienced all types of victimisation in their childhood: emotional abuse, physical abuse, emotional neglect, physical neglect, and sexual abuse. The experience of emotional neglect (*M* = 9.56; *SD* = 5.19) showed the highest incidence of child victimisation, and the other types of victimisation showed moderate rates. The analysis of the IRI confirmed high values of perspective taking (*M* = 16.70; *SD* = 4.00), empathic concern (*M* = 17.83; *SD* = 3.69), personal distress (*M* = 9.84; *SD* = 4.88), and fantasy (*M* = 13.09; *SD* = 4.39) among the participants. We found a positive and significant correlation between childhood trauma and interpersonal reactivity, specifically concerning the physical abuse (*r* = 0.22; *p*=.03) and the physical neglect (*r* = 0.24; *p*=.02) on childhood with the presence of personal distress on adulthood.

**Discussion and conclusions:**

This study pointed out the emotional neglect as the most frequent victimisation in childhood, and this result is similar to previous studies [4,5]. Contrarily to some researches [6], our sample showed high general rates of empathy on inmates. It was also possible to verify that physical abuse and physical neglect during childhood, can influence the experience of distress in those incarcerated adults. Therefore, we conclude that the traumatic events of victimisation bring negative repercussions to adult life [2].
